# Shortest Apposition Length at the First Postoperative Computed Tomography Angiography Identifies Patients at Risk for Developing a Late Type Ia Endoleak After Endovascular Aneurysm Repair

**DOI:** 10.1177/15266028221120514

**Published:** 2022-09-16

**Authors:** Anna C.M. Geraedts, Roy Zuidema, Richte C.L. Schuurmann, Ayla N. Kwant, Sana Mulay, Ron Balm, Jean-Paul P.M. de Vries, ODYSSEUS-T1EL Study Group

**Affiliations:** 1 Department of Surgery, Amsterdam University Medical Centers, Amsterdam Cardiovascular Sciences, Amsterdam, The Netherlands; 2 Department of Surgery, Division of Vascular Surgery, University Medical Center Groningen, Groningen, The Netherlands

**Keywords:** aortic aneurysm, abdominal, endovascular procedures, endoleak

## Abstract

**Purpose:**

Imaging surveillance following endovascular aneurysm repair (EVAR) is strictly recommended. This study investigates the value of endograft apposition and position relative to the aortic neck on the first postoperative computed tomography angiography (CTA) in determining patients at risk for a late type Ia endoleak (T1aEL).

**Materials and Methods:**

Patients with a T1aEL after the first postoperative CTA were selected from a consecutive database and matched with uncomplicated controls. Endograft apposition and position, including the shortest apposition length (SAL), were determined on the first postoperative CTA. The SAL is the shortest distance between the proximal endograft fabric and the first slice where circumferential apposition with the aortic wall is lost. Differences in endograft apposition at the first postoperative CTA were compared between groups. Logistic regression analysis identified independent predictors for late T1aEL.

**Results:**

A total of 32 patients with a late T1aEL were included and matched with 32 uncomplicated controls. Median follow-up after primary EVAR was 62.0 (interquartile range [IQR]: 36.8, 83.5) months in the T1aEL group compared with 47.5 (IQR: 34.0, 79.3) months in the control group; p=0.265. Median preoperative neck diameter was significantly larger in the T1aEL group than in the control group (26.6 [IQR: 24.9, 29.6] mm versus 23.4 [IQR: 22.5, 25.3] mm); p<0.001. Patients in the T1aEL group had a median SAL of 11.6 (IQR: 4.3, 20.5) mm compared with 20.7 (IQR: 13.1, 24.9) mm in the control group; p=0.002. SAL <10mm on the first postoperative CTA (odds ratio [OR]: 9.63, 95% confidence interval [CI]: 1.60–57.99) and larger neck diameter (OR: 1.80, 95% CI: 1.26–2.57) were independent predictors for developing a late T1aEL.

**Conclusion:**

Preoperative neck diameter and SAL on the first postoperative CTA following EVAR are important predictors for the development of a late T1aEL. Patients with a SAL of <10mm had a significantly higher risk of developing a late T1aEL. Future research should determine whether these patients would benefit from reintervention before an actual T1aEL is present.

**Clinical Impact:**

Understanding the mechanisms of endovascular aneurysm repair failure is essential to further enhance clinical outcomes. Adequate proximal sealing is necessary to foster freedom from type 1a endoleak. This study demonstrates that the shortest apposition length (SAL) at the first postoperative computed tomography angiography (CTA) is able to identify patients at risk for a late type 1a endoleak. Especially patients with a SAL <10mm are at high risk. Currently, the guidelines advice repeated imaging with CTA in patients with a seal <10mm. Future research should determine whether these patients would benefit from re-intervention before an actual type 1a endoleak is present.

## Introduction

Endovascular aneurysm repair (EVAR) is commonly used for elective repair of abdominal aortic aneurysms (AAA) due to the low perioperative and early mortality risk compared with open surgical repair.^1^ However, the risk of developing a late complication and the need for reintervention are considerable.^1^ Especially, endograft-related complications, such as type I and III endoleak, pose a substantial risk for late aneurysm rupture.^2^ The etiology of these endoleaks is multifactorial. Unfavorable preoperative anatomy, progressive neck dilatation, and inadequate seal between endograft and aortic wall may result in decreased proximal fixation and ultimately lead to type Ia endoleak (T1aEL) or device migration.^3–5^

To detect these complications, annual surveillance is recommended following EVAR.^6,7^ In the context of further developing surveillance protocols, a risk stratification based on the first postoperative computed tomography angiography (CTA) was proposed.^8,9^ Nevertheless, subtle changes in neck diameter and endograft position can be easily overlooked. Therefore, dedicated CTA analysis software was developed to adequately assess the shortest apposition length (SAL) of implanted endografts.^10,11^ With the aid of this software it may be possible to foresee T1aEL and migration based on changes in endograft dimensions (e.g. SAL) during follow-up.^12^ The SAL is the shortest distance between the proximal endograft fabric and the first slice where circumferential apposition with the aortic wall is lost and defines the shortest length of sealing. The aim of this study is to define the predictive value of the SAL on the first postoperative CTA after EVAR to identify patients at risk for late T1aEL. The secondary objective is to identify preoperative aortic neck characteristics that are predictors of late T1aEL.

## Materials and Methods

### Study Design

This retrospective multicenter case-control study was conducted in accordance with the STROBE guidelines and was performed in line with the Declaration of Helsinki.^13^ Patients were selected from the ODYSSEUS study cohort.^14^ The ODYSSEUS study was granted approval by the local Institutional Review Board of the Amsterdam University Medical Centres, which stated that the Dutch Medical Research Involving Human Subjects Act was not applicable. Additional approval for this study was obtained by means of an amendment for each local Institutional Review Committee.

### Patient Selection

The ODYSSEUS study included 2279 consecutive patients who underwent an elective EVAR procedure for an AAA in 1 of the 16 participating Dutch hospitals.^14^ Hospitals enrolling 5 or more patients with a late T1aEL (i.e. a T1aEL in patients without abnormalities on the first postoperative CTA) were included in the current study. The first postoperative CTA had to be made within 90 days after EVAR. Patients were excluded if no follow-up CTA was made within 90 days or if no preoperative CTA was available. CTA scans had to be acquired in the arterial phase with a slice thickness ≤3mm. Also, patients with fenestrated repair or adjunctive proximal fixation, such as endoanchors or giant stents, were excluded. T1aEL patients were matched 1:1 with uncomplicated controls based on the type of endograft and total CTA follow-up duration. The uncomplicated controls were selected from the ODYSSEUS cohort to match the complicated patients. The patients who were treated with an Endurant endograft were selected from an Endurant cohort, to effectuate comparable follow-up lengths.^15^ The total CTA follow-up length in the T1aEL group was defined as the time from EVAR until the CTA on which the endoleak was detected. Reinterventions after detection of the T1aEL are not described in this study.

### CTA Measurement Protocol

CTA measurements were performed in 3mensio, version 10.1 (Pie Medical Imaging BV, Maastricht, the Netherlands) by 2 experienced investigators (R.Z. and A.K.). A center lumen line (CLL) was automatically constructed from the celiac trunk up to the aortic bifurcation, and corrected manually if necessary. Coordinates were placed at the distal edge of the origin of the renal arteries and the lowest renal artery (LRA) was used as baseline. Neck characteristics were measured on the preoperative CTA. Neck diameter was determined from outer wall to outer wall, as the mean diameter in 2 perpendicular axes at baseline. Neck length was measured along the CLL from baseline to the point where the neck diameter increased with 10% compared with the baseline diameter. Calcification and thrombus were assessed at baseline according to the Society for Vascular Surgery (SVS) reporting standards, and the shape of the neck was determined.^16^ Straight and tapered necks were classified as non-hostile, whereas all other shapes (conical, barrel, dumbbell) were classified as hostile shape. Infrarenal angulation was defined as the angle between the longitudinal axis of the aortic neck and the longitudinal axis of the aneurysm sac. Suprarenal angulation was defined as the angle between the longitudinal axis of the suprarenal aorta and the longitudinal axis of the aortic neck. The maximum infrarenal and suprarenal curvature were calculated according to the method by Schuurmann et al.^17^ The maximum AAA diameter was determined as the mean diameter in 2 perpendicular axes and the flow lumen diameter was measured at the same slice, in the same manner. Subsequently, the flow lumen/aneurysm surface ratio was calculated using the formula for calculating the area of an ellipse (*π × minor radius × major radius*). Intended preoperative oversizing was calculated as the (*nominal endograft diameter / pre-EVAR neck diameter −* 1) *×* 100% and the effective postoperative oversizing as the *(nominal endograft diameter / post-EVAR neck diameter −* 1) × 100%.^15^ All patients were classified as inside or outside instructions for use (IFU) based on the device specific IFU with regard to the neck diameter and angulation measurements.

### Apposition Analysis

**Figure 1.**
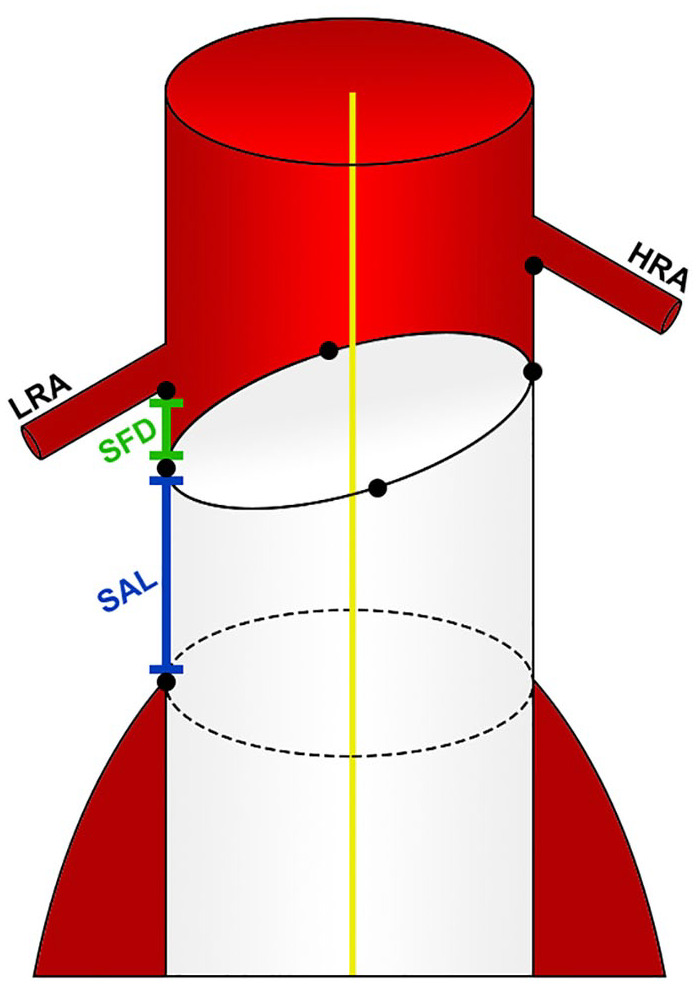
Position of the endograft in the aortic neck. The shortest apposition length (SAL) is the shortest distance between the circumference of the proximal endograft fabric and the first slice where circumferential apposition of the endograft with the aortic wall is lost. The shortest fabric distance (SFD) is the shortest distance between the endograft fabric and the lowest renal artery (LRA). HRA, highest renal artery.

The SAL and shortest fabric distance (SFD) were determined using Vascular Image Analysis (VIA) prototype software (Endovascular Diagnostics BV, Utrecht, the Netherlands), according to previously documented methods.^10,11^ In short, coordinates of the renal arteries and endograft fabric markers, and the coordinates of the slice where circumferential apposition was lost, were exported to VIA. These coordinates were combined with the CLL and a mesh of the aortic lumen. Subsequently, VIA was used to automatically calculate the 3D apposition and position of the endograft with the aortic wall. The determination of the SAL and SFD was feasible in all patients. The SAL was defined as the shortest distance between the circumference of the proximal endograft fabric and the first slice where circumferential apposition of the endograft with the aortic wall was lost. Thus, the SAL defines the shortest length of sealing along the entire circumference of the aortic neck. SFD was defined as the shortest distance between the endograft fabric and the lowest renal artery (Figure 1).^12^ The ratio of the SAL/neck length was used to assess the postoperative utilization of the preoperative neck length. A ratio of 1 equals a neck length which was completely utilized.^15^ Adequate proximal sealing of the endograft was considered in the presence of a seal length of at least 10mm. This cutoff point was based on previous literature.^8,9^

### Statistical Analysis

All data were collected in REDCap (Vanderbilt University, Nashville, Tennessee, USA), a secured electronic database. Histograms and Q-Q plots were used to assess whether continuous data were normally distributed. Not normally distributed data were expressed as medians with 25th and 75th percentiles. All characteristics and measurements were compared between the T1aEL and the control group. Differences in categorical data were tested using Fisher’s exact test and statistical differences in continuous data were assessed with the non-parametric Mann-Whitney *U* test. Binary logistic regression analyses were used to investigate the effect of a SAL <10mm on the likelihood of developing a late T1aEL, while correcting for anatomic variables (neck diameter, neck length, infrarenal angulation, and hostile shape). Results were reported as odds ratios (ORs) with the corresponding 95% confidence intervals (CI). Interaction terms for SAL <10mm were tested. A p value of 0.05 or lower was considered statistically significant. Statistical analysis was performed using IBM SPSS statistics, version 23.0 (IBM Corporation, Armonk, New York, USA).

## Results

### Baseline Characteristics

**Figure 2.**
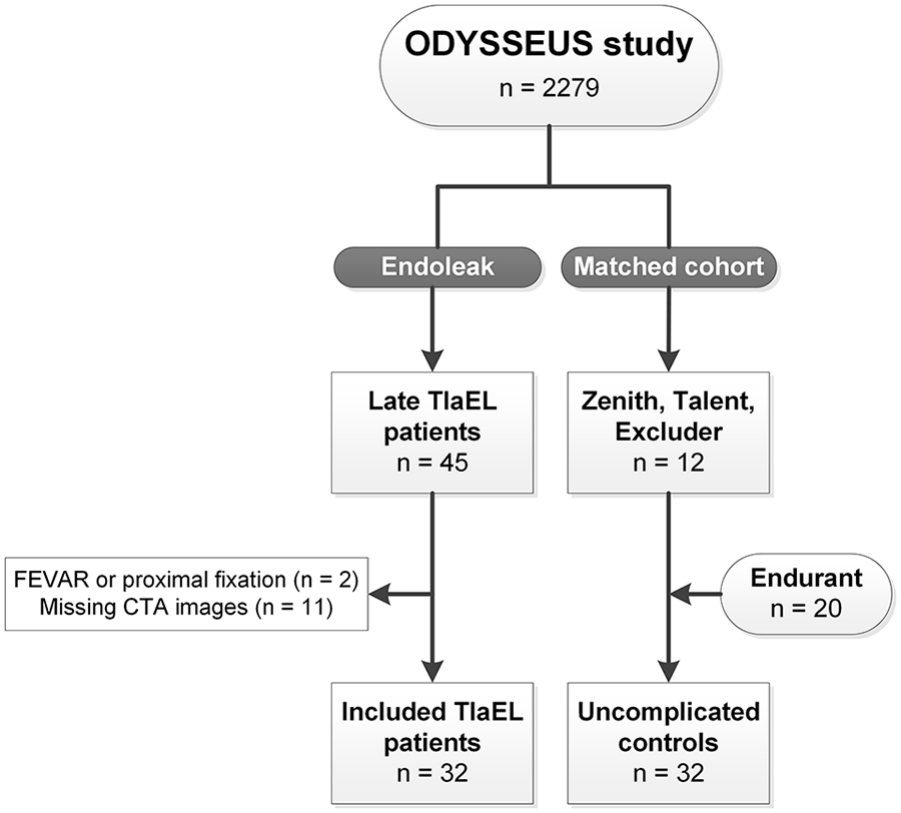
Patient selection. T1aEL, type Ia endoleak; FEVAR, fenestrated endovascular aneurysm repair; CTA, computed tomography angiography.

**Figure 3.**
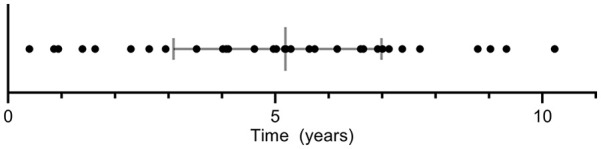
Time between endovascular aneurysm repair and computed tomography angiography with type Ia endoleak.

This study included 64 patients, 32 patients who developed a late T1aEL were matched with 32 uncomplicated controls based on type of endograft and follow-up duration (Figure 2). All patients underwent EVAR between 2007 and 2016. Table 1 provides an overview of the baseline patient characteristics. No significant differences between the groups were determined for age, sex, American Society of Anesthesiologists physical status (ASA) classification, and comorbidities. The median time between EVAR and the first postoperative CTA was 31.0 (26.0, 44.8) days in the T1aEL group and 32.5 (30.0, 42.8) days in the control group (p=0.428). The total length of CTA follow-up duration was 62.0 (36.8, 83.5) months in the T1aEL group compared with 47.5 (34.0, 79.3) months in the control group (p=0.265). The appearance of a late T1aEL ranged from 4.8 months to 10.2 years following EVAR (Figure 3).

**Table 1.** Baseline Characteristics.

	Type Ia endoleak (n=32)	Controls (n=32)	p value
Age, years	73.0 (69.0, 78.0)	69.5 (64.3, 73.8)	0.071
Male sex	28 (87.5)	28 (87.5)	1.000
ASA >2	13 (40.6)	14 (48.3)	0.611
Hypertension	18 (56.3)	19 (59.4)	1.000
Diabetes mellitus	4 (12.5)	8 (25.0)	0.337
Cardiac disease	13 (40.6)	12 (37.5)	1.000
COPD	7 (21.9)	7 (21.9)	1.000
Days until first postoperative CTA	31.0 (26.0, 44.8)	32.5 (30.0, 42.8)	0.428
Total CTA follow-up, months	62.0 (36.8, 83.5)	47.5 (34.0, 79.3)	0.265

Data are presented as median (Q1, Q3) for continuous data or n (%) for categorical data.

Abbreviations: ASA, American Society of Anesthesiologists physical status classification; COPD, chronic obstructive pulmonary disease; CTA, computed tomography angiography.

### Preoperative and Procedural Characteristics

Preoperative anatomical and procedural characteristics are presented in Table 2. In both groups, there were 20 Endurant (62.5%), 5 Zenith (15.6%), 4 Talent (12.5%), and 3 Excluder (9.4%) endografts. The neck diameter of patients in the T1aEL group was significantly larger than the neck diameter of the controls; 26.6 (24.9, 29.6) mm and 23.4 (22.5, 25.3) mm, respectively (p<0.001). Consequently, the nominal endograft diameter was significantly larger in the T1aEL group (p=0.001). There was no statistical difference in neck length between the T1aEL group and uncomplicated controls, 16.0 (9.0, 28.8) mm versus 23.6 (10.0, 30.8) mm, respectively (p=0.285). There were also no significant differences between the groups for infra- and suprarenal angulation, infra- and suprarenal curvature, thrombus, calcification, maximum aneurysm diameter, flow lumen/aneurysm ratio, intended oversizing, and accordance with IFU. There were significantly more hostile neck shapes present in the T1aEL group (p=0.005). In the T1aEL group, there were 4 straight (12.5%), 13 conical (40.6%), 7 barrel (21.9%), and 8 dumbbell (25.0%) necks. In the control group, there were 15 straight (46.9%), 11 conical (34.4%), 5 dumbbell necks (15.6%), and 1 barrel (3.1%) neck.

**Table 2.** Preoperative Anatomical and Procedural Characteristics.

	Type Ia endoleak (n=32)	Controls (n=32)	p value
Device type			
Endurant (Medtronic)	20 (62.5)	20 (62.5)	-
Zenith (Cook)	5 (15.6)	5 (15.6)	-
Talent (Medtronic)	4 (12.5)	4 (12.5)	-
Excluder (Gore)	3 (9.4)	3 (9.4)	-
Neck diameter, mm	26.6 (24.9, 29.6)	23.4 (22.5, 25.3)	**<0.001**
Endograft diameter, mm	31 (28, 36)	28 (25, 30)	**0.001**
Intended oversizing, %	11.6 (6.9, 23.4)	14.7 (8.7, 20.8)	0.658
Neck length, mm	16.0 (9.0, 28.8)	23.6 (10.0, 30.8)	0.285
Infrarenal angulation, degrees	46.5 (35.5, 61.8)	52.0 (41.0, 65.8)	0.347
Suprarenal angulation, degrees	35.5 (25.3, 60.0)	32.0 (18.0, 44.5)	0.111
Infrarenal curvature, m^-1^	37.5 (25.8, 59.0)	32.0 (23.0, 53.0)	0.600
Suprarenal curvature, m^-1^	32.0 (19.5, 46.5)	29.0 (18.3, 39.5)	0.783
Hostile shape^a^	28 (87.5)	17 (53.1)	**0.005**
Thrombus >25%	9 (28.1)	12 (37.5)	0.595
Calcification >25%	8 (25.0)	10 (31.3)	0.782
Maximum aneurysm diameter, mm	61.2 (56.5, 65.0)	59.2 (57.4, 62.6)	0.368
Flow lumen/aneurysm ratio	0.41 (0.31, 0.62)	0.33 (0.23, 0.56)	0.061
According to IFU	19 (59.4)	22 (68.8)	0.603

Data are presented as median (Q1, Q3) for continuous data or n (%) for categorical data. All statistically significant p values (≤0.05) are highlighted in bold.

Abbreviations: IFU, instructions for use.

a Hostile shape is defined as all neck shapes that are not straight or tapered.

### Postoperative Apposition

Table 3 provides an overview of the postoperative outcome measures on the first postoperative CTA. Patients with a late T1aEL had a median SAL of 11.6 (4.3, 20.5) mm compared with 20.7 (13.1, 24.9) mm in the control group (p=0.002). The SAL/neck length ratio was significantly lower in the endoleak group (p=0.017). No significant differences were observed between the groups for effective postoperative oversizing and SFD. Furthermore, 45 patients (70%) had a SAL ≥10 mm and 19 (30%) patients a SAL <10mm. When comparing these patients, a late T1aEL was seen more often in patients with a SAL <10mm (15/19; 79%) than in patients with a SAL ≥10 mm (17/45; 38%); p=0.005. These results correspond with an OR of 6.18, 95% CI: 1.76–21.71. After correcting for anatomic variables (neck diameter, neck length, infrarenal angulation, and hostile shape), multivariable logistic regression analyses identified SAL <10mm (OR: 9.63, 95% CI: 1.60–57.99) and larger neck diameter (OR: 1.80, 95% CI: 1.26–2.57) as independent predictors for T1aEL (Table 4).

**Table 3.** Postoperative Endograft Dimensions.

	Type Ia endoleak (n=32)	Controls (n=32)	p value
SAL, mm	11.6 (4.3, 20.5)	20.7 (13.1, 24.9)	**0.002**
SAL/neck length ratio	0.58 (0.20, 1.17)	0.80 (0.62, 1.46)	**0.017**
SFD, mm	1.9 (0.4, 7.1)	1.1 (0.5, 3.5)	0.320
Effective oversizing, %	9.6 (2.9, 17.1)	8.6 (1.0, 16.9)	0.643

Data are presented as median (Q1, Q3). All statistically significant p values (≤0.05) are highlighted in bold.

Abbreviations: SAL, shortest apposition length; SFD, shortest fabric distance.

**Table 4.** Univariate and Multivariable Binary Logistic Regression for Late Type Ia Endoleak.

	Univariate			Multivariate		
	OR	95% CI	p value	OR	95% CI	p value
SAL <10 mm	6.18	1.76–21.71	**0.005**	9.63	1.60–57.99	**0.013**
Neck diameter	1.49	1.17–1.89	**0.001**	1.80	1.26–2.57	**0.001**
Neck length	0.99	0.95–1.02	0.398	0.96	0.90–1.03	0.270
Infrarenal angulation	0.99	0.97–1.02	0.688	0.99	0.96–1.03	0.691
Suprarenal angulation	1.03	0.99–1.06	0.074			
Hostile shape	6.18	1.76–21.71	**0.005**	1.15	0.17–7.80	0.886
Thrombus >25%	0.65	0.23–1.87	0.426			
Calcification >25%	0.73	0.25–2.20	0.579			
Maximum aneurysm diameter	1.02	0.97–1.07	0.488			

Multivariable model is controlled for anatomical factors (i.e. neck diameter, neck length, infrarenal angulation, and hostile shape). All anatomical characteristics are measured on the preoperative computed tomography angiography (CTA) scan. SAL is measured on the first postoperative CTA scan. All statistically significant p values (≤0.05) are highlighted in bold.

Abbreviations: SAL, shortest apposition length; OR, odds ratio; CI, confidence interval.

## Discussion

This study demonstrated that EVAR patients suffering from a late T1aEL had a significantly shorter SAL on the first postoperative CTA compared with uncomplicated controls. Patients with a late T1aEL had a significantly lower SAL/aortic neck length ratio on the first postoperative CTA. Also, patients with a SAL <10 mm on the first postoperative CTA had a significant risk of developing a late T1aEL.

Late failure due to endograft-related endoleaks can lead to devastating complications, and therefore lifelong surveillance is suggested to be important.^2^ Nevertheless, there is a paradigm shift toward risk-stratified and patient-tailored follow-up protocols based on comorbidities, anatomic risk factors, and findings on the first postoperative CTA.^18^ The present study suggests that patients with a shorter SAL on the first postoperative CTA need to be identified as high risk and may benefit from intensive follow-up. This finding is partly contradictory to that of Schuurmann et al,^12^ who found no statistical difference between complicated and uncomplicated EVAR patients for the SAL on the first postoperative CTA. A possible explanation for this may be the lack of follow-up for a substantial part of their control group, which suggests that some control patients still could have developed a late T1aEL. In addition, Tripathi^19^ stated that Schuurmann et al. did not include patients from a single cohort but specifically selected high-risk patients with T1aEL who underwent reintervention in different centers.

Furthermore, this study showed that the odds of developing a late T1aEL are tenfold higher in patients with a SAL <10mm as opposed to patients with a SAL ≥10mm. The majority of patients with a SAL <10mm developed a late T1aEL (15/19; 79%). This finding is consistent with previous literature in which a risk-stratified surveillance protocol was suggested for patients based on adequate CLL seal length (>10mm) and no endoleak on the first postoperative CTA.^8,9^ They suggested to observe low risk (e.g. no endoleak and adequate seal) patients with duplex ultrasound (DUS) for the first 5 years. In patients with a CLL seal length of <10mm, the guidelines recommend repeated imaging with CTA.^6^ In addition to that, our results emphasize the importance of the measurement of SAL on the first postoperative CTA, which might be more accurate than CLL length. It should be noted that several commercially available endografts have IFU criteria including an aortic neck length of >10mm, which could potentially lead to a sealing zone of less than 10mm. This requires extra caution, especially in patients with a neck length between 10 and 15 mm in combination with other hostile neck characteristics like conicity, wide necks, and/or angulation. In these patients with a relatively short preoperative neck, the SAL/neck length ratio might be more interesting, since this represents the sealed portion of the neck. Furthermore, in these patients it might be more relevant to observe decrease of the sealing zone over time, because it is a potential risk for a type 1a endoleak.^12^

The preoperative neck characteristics showed a significantly larger neck diameter in the T1aEL group compared with the control group. This finding broadly supports other studies associating large neck diameter with delayed T1aEL, increased risk of rupture, and lower overall survival rate.^20^ As a result of this, the nominal endograft diameter was significantly larger in the T1aEL group as well. Nowadays, more complex EVAR procedures, like FEVAR or BEVAR, should be considered in patients who otherwise need an endograft with a nominal diameter of >30mm, to achieve sufficient seal in the infrarenal neck. Although this might be due to the relatively small sample size, no significant difference in neck length was observed between the two groups in this study. Previous studies have shown contradicting results regarding the relationship between preoperative neck length and developing a T1aEL.^21,22^ This inconsistency may be due to the difficulty to measure preoperative neck length in a standardized and objective manner, whereas the SAL can be seen as the actual acquired postoperative neck length. Therefore, this study advocates the possibility to replace the estimated preoperative neck length with the actual postoperative neck length, the SAL, for optimal postoperative risk stratification. The combination of the preoperative neck characteristics, with special focus on neck diameter, and postoperative SAL is advised for optimal risk stratification post-EVAR. Furthermore, no difference was observed for intended preoperative and effective postoperative oversizing between the groups. This may be explained by the fact that aortic neck dilatation is a continuous process during follow-up, and effective oversizing will decrease accordingly. Therefore, it is advised to also investigate the proximal aortic neck dilatation after EVAR, because it is related to an increased risk for late T1aEL.^3,5^

### Limitations

This study analyzed a substantial amount of late T1aEL patients that were available from one multi-center cohort study. By doing so, it was possible to gain insight in the preoperative and postoperative characteristics of late T1aEL patients. Despite the relatively large number of T1aEL patients in this study, the overall sample size remains small. Therefore, the findings must be interpreted with caution and might be considered hypothesis generating. Moreover, to a certain extent there is almost inevitable selection bias for the matched control group. To counter this, the controls were matched 1:1 on endograft type and follow-up duration. The use of a larger uncomplicated control group could have increased the reliability of the findings. Unfortunately, it turned out to be challenging to find uncomplicated controls with comparably long CTA follow-up for all type of endografts. Therefore, we chose 1:1 matching since this would result in a comparable control group, instead of a larger control group without matched follow-up length. To effectuate comparable follow-up, it was already necessary to include patients with an Endurant endograft in the control group from another cohort, which consisted of patients that were certainly uncomplicated and already analyzed. Another limitation is that the VIA software used to calculate the SAL is not yet Conformité Européenne (CE) marked, although this process is underway. Therefore, implementing the SAL in the clinical practice is not yet possible. Our advice would be to use the shortest apposition distance along the CLL as an alternative until the VIA software is CE-approved and available.

## Conclusion

The determination of the shortest apposition length on the first postoperative CTA following EVAR is of major importance to identify patients at risk for late failure. Patients with a SAL of <10mm and a larger aortic diameter had a significant risk of developing a late T1aEL. These findings need further evaluation. Currently, the guidelines advice repeated imaging with CTA in patients with a seal <10mm. Future research should determine whether these patients would benefit from reintervention before an actual T1aEL is present.
